# Effect of cAMP Receptor Protein Gene on Growth Characteristics and Stress Resistance of *Haemophilus parasuis* Serovar 5

**DOI:** 10.3389/fcimb.2020.00019

**Published:** 2020-02-25

**Authors:** Changsheng Jiang, Yufang Cheng, Hua Cao, Bingzhou Zhang, Jing Li, Ling Zhu, Zhonghua Li, Wei Zeng, Chang Li, Qigai He

**Affiliations:** ^1^State Key Laboratory of Agricultural Microbiology, College of Animal Sciences and Veterinary Medicine, Huazhong Agricultural University, Wuhan, China; ^2^The Cooperative Innovation Center for Sustainable Pig Production, Wuhan, China

**Keywords:** *Haemophilus parasuis*, *crp* gene, cAMP receptor protein, growth characteristics, stress resistance, biofilm formation

## Abstract

*Haemophilus parasuis* (HPS), a member of the family Pasteurellaceae, is a common bacteria in the upper respiratory tract of pigs but under certain circumstances can cause serious systemic disease (Glasser's disease) characterized by severe infection of the upper respiratory tract, fibrinous polyserositis, polyarthritis, and meningitis. cAMP receptor protein (CRP) is among the most important global regulators, playing a vital role in adapting to environmental changes during the process of bacterial infection. In order to investigate the function of the *crp* gene in the growth characteristics of *H. parasuis* serovar 5 (HPS5) and its ability to overcome adverse environmental stresses, a *crp* mutant strain (Δcrp) was constructed and verified. In this study, we found that the *crp* gene was involved in growth rate, biofilm formation, stress tolerance, serum resistance, and iron utilization. Compared with the wild type, both the growth rate of the *crp* mutant and its resistance to osmotic pressure decreased significantly. Similar phenomena were also found in biofilm formation and iron utilization. However, the resistance to heat shock and serum complement of the *crp* mutant were enhanced. This study aimed to reveal the function in growth characteristics and stress resistance of the *crp* gene in HPS5. Whether it relates to virulence requires additional in-depth research.

## Introduction

*Haemophilus parasuis* is a member of the family Pasteurellaceae, the growth of which is strictly dependent on V factor (nicotinamide adenine dinucleotide, NAD) but not X factor (hemin) (Biberstein and White, 1969; Jin et al., 2006). *H. parasuis* is a common bacteria that existed in the upper respiratory tract of pigs, but under specific circumstances, such as hypoimmunity, stress, poor feeding, and management practices, etc., it can cause serious systemic disease (Glasser's disease). Glasser's disease is characterized by severe infection of the upper respiratory tract, fibrinous polyserositis, polyarthritis, and meningitis in pigs, which leads to the huge economic losses in the global pig industry (Liu et al., 2016). However, the pathogenic mechanism is not well-understood. *H. parasuis* could be classified into at least 15 serotypes. In general, serotypes 1, 5, 10, 12, 13, and 14 are considered as highly virulent serovars; 2, 4, and 15 as moderately virulent; with serovars 3, 6, 7, 8, 9, and 11 regarded as non-virulent (Jin et al., 2006; Zhang et al., 2019). In China, the most prevalent serotypes are serovars 4 and 5, followed by 13, 14, and 12; 12% of isolates are non-typable (Cai et al., 2005).

cAMP receptor protein (CRP) is one of seven global regulators in *Escherichia coli*, which can regulate ~490 genes (Geng and Jiang, 2015). Global regulators play a vital role in the adaption of bacteria to environmental changes during the infection process. Previous studies have demonstrated that CRP could improve the performance of *E. coli* under various stressful conditions, including osmotic pressure, bioalcohol stress, oxidative stress, low pH, and in acetate and organic solvents (Zhang et al., 2012c; Basak et al., 2014; Geng and Jiang, 2015). CRP is the first prokaryotic transcription factor purified and crystallized from *E. coli* and also the most comprehensively characterized regulator (Emmer et al., 1970; McKay and Steitz, 1981). This protein modulates the expression of multiple genes in response to changes in intracellular concentrations of cAMP (Gosset et al., 2004; Zhao et al., 2016), which is synthetized by adenylate cyclase. When cAMP binds to CRP, the cAMP–CRP complex changes the conformation and binds to promoters containing the consensus sequence TGTGAN_6_TCACA (Zhao et al., 2016). After binding to the promoter, CRP can recruit RNA polymerase and initiate transcription of the target gene. With some promoters, CRP also inhibits transcription through several mechanisms, such as promoter occlusion (Zhao et al., 2016). By mutagenesis, it has been confirmed that CRP is related to the expression of multiple virulence factors. The virulence of many gram-negative bacteria, including *Edwardsiella piscicida* (Choe et al., 2017), *Salmonella* (Chen et al., 2010), *Vibrio cholera* (Zahid et al., 2015), and *Yersinia enterocolitica* (Petersen and Young, 2002) is attenuated by deletion of the *crp* gene. Since the *crp* gene is closely related to virulence, it has usually been selected as a target for the development of attenuated vaccines (Kelly et al., 1992; Hassan and Curtiss, 1994; Desin et al., 2013).

The function of the *crp* gene in *H. parasuis* has so far not been identified. Therefore, in the present study, the functions of the *crp* gene in HPS5 were investigated by the comparison of growth characteristics, the ability to undergo autoagglutination, biofilm formation, stress and serum resistance, and iron utilization of wild-type and *crp* mutant strains. Whether this gene is related to the virulence of *H. parasuis* requires additional study.

## Materials and Methods

### Bacterial Strains, Plasmids, and Culture Conditions

The bacterial strains and plasmids used in this study are listed in Table 1. The standard reference strain of *H. parasuis* serotype 5 (HPS5) was cultured in Tryptic Soy Broth (TSB) or Tryptic Soy Agar (TSA) medium (Difco Laboratories, Detroit, MI, USA) supplemented with 10 μg/ml NAD and 8% (v/v) inactivated cattle serum (T/V/S) (Zhejiang Tianhang Biotechnology, Zhejiang, China) at 37°C. The culture conditions of the mutant strain (Δcrp) were the same as those for the wild type but with additional kanamycin (50 μg/ml) (Sigma-Aldrich, Missouri, USA). *E. coli* DH5α was cultured in TSA or Luria–Bertani medium at 37°C.

**Table 1:** The strains and plasmids used in this study.

**Strains and plasmids**	**Characteristics**	**Source/References**
**STRAINS** *Escherichia coli* DH5α	Cloning host for maintenance of recombinant plasmids	Purchased from TaKaRa (Otsu, Japan)
*Haemophilus parasuis* 5 (HPS5)	Reference strain of serotype 5	Preserved in our lab
*H. parasuis* Δ*crp::kan* (Δ*crp*)	*Crp* mutant strain, Kan resistance	This study
**PLASMIDS** pK18mobsacB	Suicide and narrow-broad-host vector, Kan resistance	Zhang et al., 2012b
pK18-Δcrp::kan	A 2,039 bp overlap fragment containing Kan, the upstream and downstream sequences of the crp gene in pK18mobsacB, Kan resistance	This study
pSHK3	*E. coli–H. parasuis* shuttle vector, Kan resistance	Wang et al., 2013

### Construction and Verification of *crp* Mutant Strain

All plasmids and primers used for the construction of the *crp* mutant strain are listed in the Tables 1, 2. The upstream (565 bp) and downstream (565 bp) fragments of the *crp* gene were amplified from the HPS5 genome using primer pairs crp-uF/uR and crp-dF/dR, respectively. And the kanamycin resistance cassette gene (909 bp) was amplified from pSHK3 plasmid with the primer Kan-F/R. These three fragments were linked with overlap extension PCR using the primer crp-uF/dR to construct a new fragment UKD (*crp* upstream sequence, kanamycin resistance cassette sequence, and *crp* downstream sequence). The UKD fragment obtained in this way was then inserted into a pk18mobsacB plasmid with *BamHI* and *SalI* restriction enzymes to generate the recombinant plasmid pk18-crp-UKD. The recombinant plasmid was introduced into HPS5 by the nature transformation method as previously described (Zhang et al., 2012b, 2019; Wang et al., 2013) with some modifications. Briefly, 20 μl of cAMP (8 mM) was added to 20 μl wild-type suspension in logarithmic phase (OD_600_ value at 0.9). Ten minutes after the reaction at room temperature, 2 μg of the recombinant plasmid pk18-crp-UKD was added to the bacterial suspension, mixed, and reacted for another 10 min. The cells were then added to a T/V/S plate and incubated at 37°C for 6 h. Subsequently, bacteria were transferred to a kanamycin selective plate. Finally, the cells were incubated at 37°C for 24–48 h. To verify the construction results of the *crp* mutant, the UKD sequence, kanamycin resistance cassette gene, and *crp* gene were amplified and then verified by sequencing.

**Table 2:** The primers used to construct and verify the crp mutant.

**Primers**	**Characteristics and sequence**	**Source**
crp-uF/uR	CGCGGATCCACCGCTTGTACAGGACATGCATTAATGTT; TTATCTTGTGCAATGAGAAACCTCTATAAATCATTTA; to amplify the upstream fragment of *crp*, 565 bp	This study
Kan-F/R	TAAATGATTTATAGAGGTTTCTCATTGCACAAGATAA; GCACTTTGCATTTTTAATATGCAATTAACCAATTCTGATTAG; to amplify sequence of kanamycin resistance gene, 909 bp	This study
crp-dF/dR	CTAATCAGAATTGGTTAATTGCATATTAAAAATGCAAAGTGC; ACGCGTCGACACAAGCGGTCGCGGCAATAGAAATCACTC; to amplify the downstream fragment of *crp*, 565 bp	This study
crp-F/R	ATGCAAGATGTTTCAATCTCAACCG; TTATCTTGTCCCATACAC; to amplify sequence of *crp* gene, 675 bp	This study

### The Growth Characteristics of Two *H. parasuis* Strains

The growth characteristics of the wild-type and *crp* mutant strains were measured (Wang et al., 2017; Zhang et al., 2019). The wild-type and *crp* mutant strains were cultured in 5 ml fresh T/V/S medium overnight and then diluted with the same medium until the OD_600_ value had reached 0.4. A 100 μl volume of diluted cell suspension was added into 100 ml fresh T/V/S media and incubated in a shaker at 180 rpm at 37°C for 24 h. The OD_600_ value of each culture was measured at 2 h intervals using an Eppendorf Biospectrometer (Eppendorf, Hamburg, Germany). The number of colony forming units (CFUs) was measured by serial dilution and plating at 4 h intervals. The experiments were performed in triplicate three times.

### Autoagglutination Assay

The autoagglutination ability of HPS5 and Δcrp was determined as described in previous studies with some modifications (Huang et al., 2016; Zhang et al., 2019). HPS5 and Δcrp were grown in 15 ml T/V/S medium overnight and cultured to stationary phase and then transferred into 15 ml sterile tubes, and the OD_600_ value was adjusted to ~0.8. The tubes were maintained statically at 37°C for 60 h. At 0, 3, 6, 9, 12, 24, 36, 48, and 60 h, 200 μl medium was carefully collected from the top of the culture, and the OD_600_ was measured. The experiments were repeated three times with triplicated readings.

### Biofilm Formation Assay

A biofilm formation assay was conducted in 96-well flat-bottomed microtiter plates (Thermofisher, USA) as described in previous studies with some modifications (Zhang et al., 2019). Briefly, for the wild-type strain, overnight cultures were collected and diluted with fresh medium to an OD_600_ of 0.8. For the *crp* mutant, 2 ml of overnight culture was harvested by centrifugation at 5,000 rpm and resuspension in 1 ml fresh medium, and then diluted to an OD_600_ value of 0.8. The 20 μl of diluted bacterial solution was added to each well-containing 180 μl fresh T/V/S medium and then statically incubated at 37°C for different durations (12, 24, 36, and 48 h). Each strain was tested in triplicate. To expose each biofilm, the liquid fraction from each well was removed with an injector, and then the wells were washed three times with 200 μl sterile PBS to remove loosely adherent cells. The remaining bacteria attached to the wells were fixed with 100 μl methanol for 30 min. After air drying, the wells were stained with 200 μl of 1% crystal violet solution for 10 min at room temperature. Excess crystal violet was removed from the wells by placing the plate under running tap water until waste liquid had clarified. Thereafter, the plates were dried in a 37°C incubator for 30 min and the attached cells dissolved in 100 μl of 33% (v/v) glacial acetic acid, and then the OD_630_ values were measured using a Synergy™ HT Multi-Detection Reader (Bio Tek Instruments, USA). All tests were conducted in sextuplicate three times, and the mean of results was recorded. The wells containing only 200 μl fresh T/V/S medium were used as negative controls.

### Stress Resistance Assay

Stress resistance assays were performed based on a previously described method (Huang et al., 2016) with some modifications. Briefly, the OD_600_ values of cultures of wild-type and *crp* mutant strains incubated overnight were adjusted to 0.8. For the osmotic stress tolerance assay, 100 μl of 100 mM potassium chloride was added to 900 μl of cell suspension and incubated at 37°C for 30 and 60 min. In the heat shock assay, 1 ml of the diluted bacterial suspension was incubated in a 45°C water bath for 30 and 60 min. In the oxidative stress tolerance assay, 100 μl of 1 M hydrogen peroxide was added to 900 μl of cell suspension, which was incubated at 37°C for 30 and 60 min. Untreated cell suspensions of each strain after incubation at 37°C for each duration represented controls for each experiment. After incubation, the cultures were serially diluted in PBS, and CFUs were measured by plate counting. The percentage of stress-resistant cells was calculated as [(stressed sample CFU/ml)/(control sample CFU/ml)] × 100%. Each assay was performed independently three times.

### Sera and Serum Bactericidal Assay

Normal swine serum was obtained from the Cooperative Innovation Center for Sustainable Pig Production in Wuhan, China. The porcine serum was collected from six healthy pigs (1.5–2 months old) from a farm free of Glasser's disease. Serum was filter-sterilized (0.22 μm) and stored at −80°C until used. Some aliquots of the sera were treated at 56°C for 30 min to inactivate the complement.

The serum bactericidal assay was performed with porcine serum as previously described (Zhang et al., 2012a) with some modifications. For the 50% serum bactericidal assay, 100 μl of bacterial suspension (~1 × 10^8^ CFU/ml) was mixed with 100 μl of either fresh serum or inactivated swine serum to achieve a final concentration of 50% serum. For the 90% serum bactericidal assay, a 180 μl aliquot of either fresh or heat-treated serum was mixed with 20 μl of bacterial suspension (~1 × 10^7^ CFU/ml) to achieve a final concentration of 90% serum. The mixtures were incubated at 37°C for 1 h. After incubation, they were then 10-fold serially diluted and transferred to T/V/S plates, and continue to incubate under the same conditions for 24 h. The number of colonies was then recorded. The proportion of colonies surviving was calculated from the ratio of the number in fresh serum to those in heat-treated serum. Each experiment was repeated three times, independently.

### Iron Utilization Assay

The ability of wild-type and *crp* mutant strains to utilize iron was measured as previously described (He et al., 2018; Dong et al., 2019) with some modifications. Briefly, the two strains were cultured to log-phase replication, and the value of OD_600_ of the cultures was adjusted to 0.4. Then 50 μl volume of diluted cell suspension was added to 5 ml fresh T/V/S medium supplemented with 100 μM 2,2-bipyridyl (BIP, Sigma, USA) to cause iron restriction, or 100 μM FeSO_4_ was added into the iron restriction medium as the iron source, respectively, using fresh normal T/V/S medium (0 μM FeSO_4_) as the control. Strains were grown at 37°C with shaking for 12 h, after which the OD_600_ was measured. The experiments were repeated three times independently and the means of results recorded.

### Real-Time PCR

Wild-type and *crp* mutant strains were cultured overnight, and then total RNAs were extracted using Trizol reagent (Invitrogen, Carlsbad, CA, USA), in accordance with the manufacturer's instructions. For each sample, cDNAs were synthesized with the PrimeScript™ RT reagent Kit (TaKaRa, Dalian, China). Real-time PCR was performed with a ViiA™ 7 Real-Time PCR system using Power SYBR Green PCR Master Mix (Applied Biosystems, USA). 16S rRNA was amplified as an endogenous control, and the results were analyzed using the 2^ΔΔCT^ method in triplicate in three independent experiments. The primers used for real-time PCR are listed in Table 3.

**Table 3:** The primers used for real-time PCR.

**Primers**	**Sequences**
tbpA-F	ACTTACCGCTTGAATGGCGA
tbpA-R	CGTCCAACGGTGCTAGTTCT
tbpB-F	GGCAGGTAGCGGGTTTACAA
tbpB-R	GTTGCAGTACGTTCGCCTTG
cirA-F	TGGTGGAAACGACCGCATTA
cirA-R	TCAGCGTGACCACGATCAAA
16S-F	AAGAAGCACCGGCTAACTCC
16S-R	CGGGGCTTTCACATCTCACT

### Statistical Analysis

The results are presented as means ± standard deviation (SD). Results were evaluated by analysis of multiple *t*-test in GraphPad Prism 7.0 (GraphPad Software Inc., USA). *P* < 0.05 was considered statistically significant (^*^), while *p* < 0.01 was regarded as highly significant (^**^).

## Results

### Construction and Verification of crp Mutant Strain

**Figure 1:**
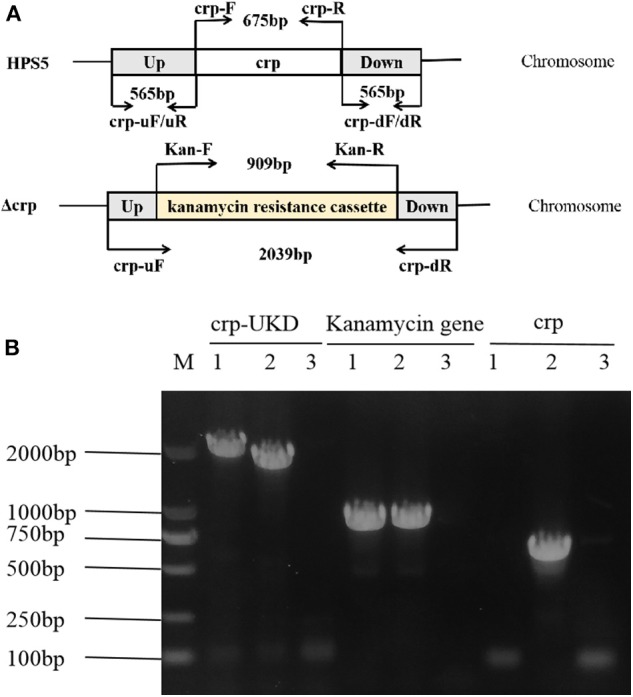
Construction and verification of the *crp* mutant strain. The primers for amplification of specific target fragments were labeled. The primer pair crp-uF/dR was used to detect the UKD sequence, Kan-F/R to detect the kanamycin cassette, and crp-F/R to detect the *crp* gene. **(A)** PCR identification of the *crp* mutant strain by amplification of the crp-UKD (2,039 bp), kanamycin resistance cassette sequence (909 bp), and *crp* gene (675 bp) fragments. **(B)** M: DL 2000 Mark, lane 1: Δcrp genome, lane 2: positive control [*Haemophilus parasuis* serovar 5 (HPS5) genome or pSHK3 plasmid], and lane 3: negative control (DNase/RNase free water).

The *crp* mutant strain of HPS5 (Δcrp) was constructed by homologous recombination. And the results of PCR verified that it was successfully constructed. As shown in Figure 1A, the primer pairs crp-uF/dR, Kan-F/R, and crp-F/R were used to detect the UKD sequence (2,039 bp), the kanamycin resistance cassette sequence (909 bp), and the *crp* gene (675 bp), respectively. In the *crp* mutant, both the UKD sequence (2,039 bp), which was a little larger than that of the wild type (1,805 bp), and the kanamycin resistance cassette sequence were able to be amplified, while the *crp* gene could not (Figure 1B). These results indicate that the *crp* mutant was successfully constructed. The same result was obtained in sequencing.

### Growth Characteristics of HPS5 and Δcrp

**Figure 2:**
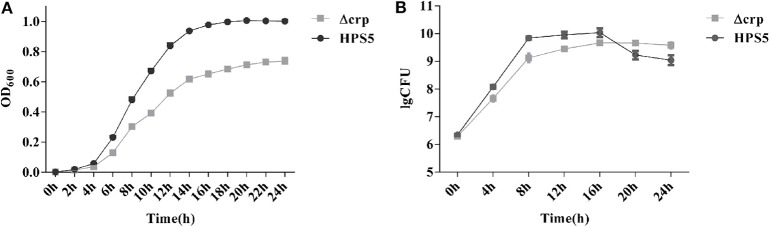
Growth analysis of the wild type (HPS5) and the *crp* mutant (Δcrp). Overnight cultures were diluted in T/V/S medium and then incubated in a shaker at 180 rpm at 37°C for 24 h **(A,B)**. Bacterial growth was monitored by measurement of optical density at 600 nm **(A)** and by viable cell counts in log CFU (colony forming unit)/ml **(B)** at multiple time points. Data points and error bars represent mean values of three replicates and standard deviations, respectively.

The growth characteristics of the HPS5 and Δcrp strains were investigated at 37°C. The results indicated that the growth characteristics of the two strains were significantly different. Compared with the wild type, the growth of Δcrp was clearly slower. The largest OD_600_ value of the wild type was ~1.0, while that of the Δcrp strain was only ~0.7. In addition, the HPS5 required ~16 h to reach the stationary phase and remained relatively stable, while the Δcrp strain required ~14 h (Figure 2A), but after 14 h, the OD_600_ value of Δcrp still increased slowly, until 22 h from the start of the experiment.

The observed numbers of bacteria suggested that the two strains also exhibited significant differences. Before the 16 h time point, the number of CFUs of Δcrp was significantly lower than that of the wild type. By the 16 h time point, the number of CFUs of the two strains reached their maximum: 1.09 × 10^10^ CFU/ml for the wild type and 4.65 × 10^9^ CFU/ml for the *crp* mutant strain. However, between 20 and 24 h, the number of Δcrp strain CFUs was significantly higher than the number of HPS5. After 16 h, the wild type reached its stationary phase, and the number of living bacteria had decreased, but at the same time, the Δcrp strain continued to grow slowly, so the number of living Δcrp bacteria was ultimately higher than HPS5 (Figure 2B). These results indicated that the growth of the *crp* mutant strain was slower than the wild type.

### Autoagglutination Capability of HPS5 and Δcrp

**Figure 3:**
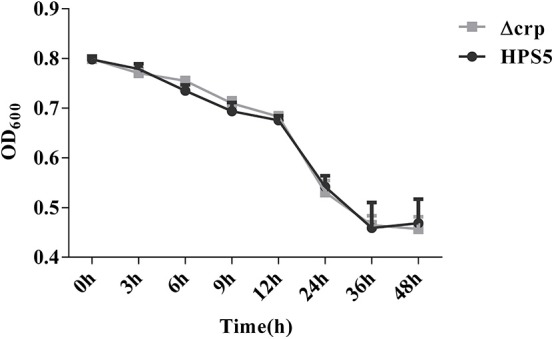
Autoagglutination capability of HPS5 and Δcrp strains at 37°C. Overnight cultured bacteria were diluted such that the OD_600_ value was close to 0.8. An aliquot of 0.2 ml from the top of the culture was removed to measure the OD_600_ value at 3, 6, 9, 12, 24, 36, and 48 h post-inoculation at 37°C. All of the above points and error bars represent the mean value of three replicates and standard deviations, respectively.

Autoagglutination appears to be a virulence-associated trait in number of gram-negative bacteria (Zou et al., 2013). In the present study, the autoagglutination capability of HPS5 and Δcrp was measured at 37°C. Compared with HPS5, Δcrp failed to exhibit a reduction in autoagglutination capability (Figure 3). These results indicated that *crp* gene deletion of HPS5 had no impact on autoagglutination.

### Biofilm Formation Capability Decreased in Δcrp

**Figure 4:**
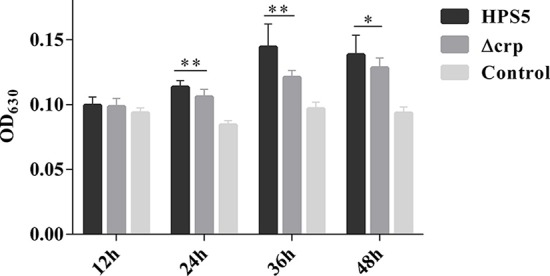
Biofilm formation capability of the HPS5 and Δcrp strains at different time points. Biofilm formation was determined by measuring the OD_630_ value of dissolved crystal violet at 12, 24, 36, and 48 h post-incubation. Each strain was tested in 6 wells in a 96-well microtiter plate. All the assays above were performed in triplicate. Bars represent the means ± standard deviation of three independent experiments. Statistical analyses were performed using the multiple *t*-test. *p < 0.05; **p < 0.01.

The ability of HPS5 and Δcrp to form biofilms was compared in polystyrene microtiter plates using crystal violet staining, followed by quantitative analysis using a microplate reader. The results indicated that no formation of biofilm was observed in the HPS5 and Δcrp strains at the start of the first 12 h period. However, compared with the wild type, the ability of the *crp* mutant strain to form a biofilm was significantly decreased at 24, 36, and 48 h (*p* < 0.05 or 0.01, *t*-test) (Figure 4). These results suggested that *crp* gene deletion in HPS5 decreased the ability of the bacteria to form biofilms.

### Stress Resistance Results

**Figure 5:**
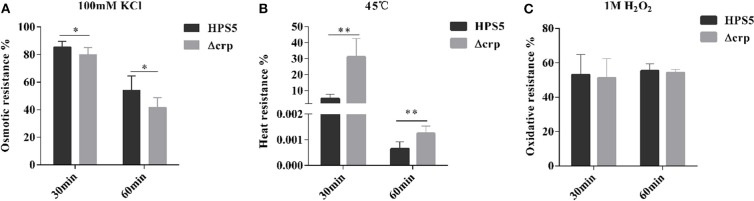
Analysis of the stress tolerance of the HPS5 and Δcrp strains. Overnight cultures were adjusted to an OD_600_ value of 0.8. A total of 900 ml of each cell suspension was then treated **(A)** with 100 μl of 100 mM potassium chloride, **(B)** by incubation in a 45°C water bath, or **(C)** with 100 ml of 1 M hydrogen peroxide for 30 and 60 min. * and ** denote *p*-values (*t*-test) of <0.05 and <0.01.

HPS5 and Δcrp strains were exposed to different stress conditions, including osmotic pressure, oxidative stress, and heat shock. When bacterial cells were evaluated with 100 mM potassium chloride for 30 and 60 min, the survival rates of HPS5 were 85.41 and 53.93%, respectively, which were significantly higher than the survival rate of Δcrp (79.93 and 41.71%, respectively) (*p* < 0.05, *t*-test) (Figure 5A). The opposite trend was observed in the heat shock assay. When the bacterial strains were incubated in a 45°C water bath for 30 and 60 min, the survival rates of Δcrp (31.16 and 0.0012%) were significantly higher than those of the wild type (5.15 and 0.0006%) (*p* < 0.01, *t*-test) (Figure 5B). However, when the cells were treated with 1 M hydrogen peroxide for 30 and 60 min, HPS5 and Δcrp exhibited similar survival rates (52.98 and 51.18%, and 55.40 and 54.26%, respectively) (*p* > 0.05, *t*-test) (Figure 5C). These results indicated that the *crp* gene was able to increase the tolerance of osmotic stress, but it decreased the tolerance of heat shock and did not affect the tolerance of HPS5 to oxidative stress.

### Wild-Type Strain Exhibited Greater Sensitivity to Complement

**Figure 6:**
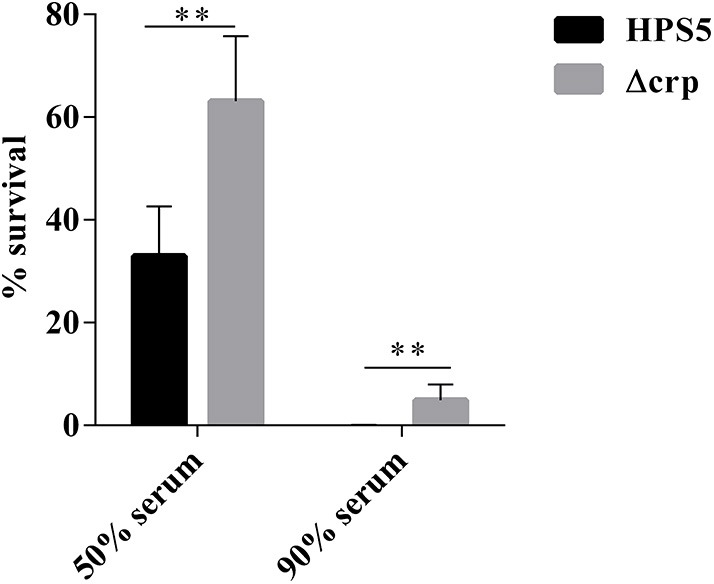
Analysis of survival of the HPS5 and Δcrp strains in 50 and 90% swine serum. For the 50% serum bactericidal assay, 100 μl of bacterial suspension (~1 × 10^8^ CFU/ml) was mixed with 100 μl of either fresh or inactivated swine serum. For the 90% serum bactericidal assay, 20 μl of bacterial suspension (~1 × 10^7^ CFU/ml) was mixed with 180 μL of either fresh or inactivated swine serum and then incubated at 37°C for 1 h. The proportion of bacteria surviving was calculated by the ratio of colonies in fresh serum to those in heat-treated serum. **p < 0.01.

We investigated the effect on serum resistance of the wild type and *crp* mutant in 50 and 90% swine serum. The results indicated that the survival rate of the *crp* mutant in 50% swine serum was 63.11%, significantly greater than that of the wild type (32.9%) (*p* < 0.01, *t*-test). Similar results were observed in the 90% swine serum resistance assay. Survival rates of the wild-type and *crp* mutant strains were 0.05 and 4.91%, respectively, indicating that Δcrp exhibited significantly greater survival HPS5 (*p* < 0.01, *t*-test) (Figure 6). These results showed that the wild type was more sensitive to the effects of complement.

### Iron Utilization Results

**Figure 7:**
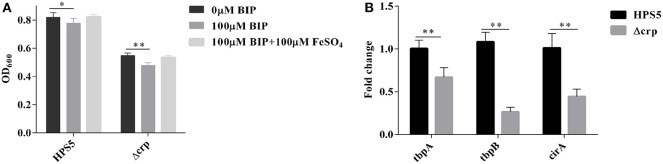
The capability of iron utilization in the HPS5 and Δcrp strains. Overnight cultures were diluted such that they had an OD_600_ value of 0.4. Growth of the HPS5 and Δcrp strains in the iron-restricted medium and iron-supplemented medium and monitored by measurement of optical density at 600 nm after 12 h incubation. **(A)** Real-time PCR detected the expression of tbpA, tbpB, and cirA, each related to iron uptake. 16S rRNA was used as the endogenous control. **(B)** Error bars represent the standard deviations of three independent experiments. * and ** denote *p*-values (*t*-test) of <0.05 and <0.01, respectively.

To ascertain the ability of the *crp* gene in HPS5 to utilize iron, the growth characteristics of the HPS5 and Δcrp strains were tested in iron-restricted medium with 100 μM BIP or supplemented with 100 μM FeSO_4_ as the source of iron, respectively. The results indicated that the growth of HPS5 decreased slightly (*p* < 0.05, *t*-test) when exposed to the 100 μM BIP, but the growth of the *crp* mutant strain decreased significantly (*p* < 0.01, *t*-test). When supplemented with 100 μM FeSO_4_ in iron-restricted medium, the growth rate of both the wild-type and *crp* mutant strains were restored (Figure 7A). Real-time PCR confirmed that the expressions of tbpA, tbpB, and cirA, each related to iron uptake, in the *crp* mutant were significantly downregulated compared with the wild type (Figure 7B). Therefore, the *crp* mutant was more sensitive to an iron restriction environment. These results indicated that the *crp* gene in HPS5 may be involved in the utilization of sources of iron.

## Discussion

Global regulators play a vital role in adapting bacteria to environmental changes during the process of infection. CRP is one of seven global regulators in *E. coli*, which play important roles in adapting to environments and regulating virulence. However, so far no report has been published that illustrates the function of *crp* in *H. parasuis*. In order to explore the function of the *crp* gene in *H. parasuis*, a *crp* mutant strain of HPS5 was constructed, and the polarity effect assay (Supplementary Table 1 and Figure S1) verified the change in function of the Δcrp strain was caused by *crp* gene rather than any other upstream or downstream genes. We analyzed the role of the *crp* gene of HPS5 in terms of growth characteristics and stress resistance. The results indicated that the growth rate and number of living bacteria in the *crp* mutant strain decreased significantly compared with the wild-type reference strain during its stationary phase (Figures 2A,B) as observed with *Klebsiella pneumoniae* (Ou et al., 2017), *Vibrio vulnificus* (Kim et al., 2013), and avian *Pasteurella multocida* (Zhao et al., 2016). We not only demonstrated that the *crp* gene of HPS5 affected the growth of the strain but also found that it played a role in its ability to autoagglutinate. The results indicated that the *crp* gene did not affect the autoagglutination capability of HPS5 and Δcrp (Figure 3), but the capability decreased in the *crp* mutant of *K. pneumoniae* serotype K1 (Ou et al., 2017). This may be due to the specific attributes of different species. Glasser's disease can cause serious systemic morbidity characterized by severe infection of the upper respiratory tract, fibrinous polyserositis, polyarthritis, and meningitis in pigs. The living environment of bacteria can be changed by severe inflammatory reactions, including osmotic pressure, oxidative stress, and heat shock. These changes may affect the viability of the bacteria. Previous studies have established that CRP can indirectly mediate the expression of a large number of cytoplasmic stress response proteins, including major chaperons, five ATP-dependent protease complexes, heat shock proteins, etc. (Gosset et al., 2004). In this study, stress resistance assays evaluated the survival of the bacterial strains within a variety of stress conditions. The results indicated that the *crp* gene increased the tolerance of the bacteria to osmosis but decreased their tolerance to heat shock and had no effect on the tolerance of HPS5 to oxidative stress (Figures 5A–C). However, the *crp* gene in *E. coli* has been shown to increase the tolerance to high osmotic pressure and provide oxidative resistance (Basak and Jiang, 2012). These differences may be caused by different levels of stress response protein expressed in different bacteria.

Biofilm formation is a complex multi-factorial process in many bacteria. Firstly, bacterial cells attach to the surface of a polymer, produce a slimy extracellular matrix to mediate cell-to-cell adherence, and then finally produce multilayered cell clusters (Costerton et al., 2005). Biofilm formation plays an important role in bacterial adaption to environmental change (Joo and Otto, 2012), which can help *H. parasuis* to colonize the upper respiratory tract of pigs over a long duration. Furthermore, biofilms play an important role in immune escape and tolerance of antimicrobial agents, leading to persistent and chronic infection (Jin et al., 2006). A previous study confirmed that most serovars of *H. parasuis* strains can form biofilms *in vitro* (Jin et al., 2006). Formation of a biofilm from *K. pneumoniae* serotype K1 has been shown to be impaired following deletion of the *crp* gene (Ou et al., 2017). In the present study, the capability of Δcrp to form biofilms was clearly less than that of the wild type (Figure 4). The results indicated that the *crp* gene plays an important role in biofilm formation in HPS5 and that *crp* gene mutation may decrease the ability of the bacteria to adapt to environmental changes.

The ability of bacteria to cause systemic infection often corresponds to its resistance to bactericidal activity of host complement proteins, which can allow bacteria to effectively escape from the host immune response and to survive in the blood stream (Cerdà-Cuéllar and Aragon, 2008). Therefore, the serum resistance represents an important virulence strategy of bacterial pathogens. The most interesting results are that the serum bactericidal assays demonstrated that survival of the *crp* mutant in 50 and 90% swine serum increased significantly (Figure 6) compared with the wild-type strain. These results indicated that the *crp* gene can reduce the ability of HPS5 to exhibit serum resistance. However, the serum resistance of *K. pneumoniae* serotype K1 did not exhibit any clear difference when the *crp* gene was deleted (Ou et al., 2017). Several hypotheses concerning the mechanism of this serum resistance have been proposed, such as decreased binding of IgG or increased binding of host complement alternative pathway inhibitor, fH (Wang et al., 2018). Thus, we hypothesize that the difference may be caused by a variation in the ability of the *crp* gene to bind IgG or fH in different bacteria.

Iron is required for bacteria to colonize host tissues. It plays a key role in enzymatic activity and metabolic processes including electron and oxygen transportation (Imlay, 2008; Roux et al., 2009). Iron may also regulate the expression of multiple virulence factors, such as biofilm formation, motility, and invasion (Hsu et al., 2018). Iron is a double-edged sword for bacteria. On one hand, excessive iron results in iron toxicity to cellular components, in particular, damage to DNA (Halliwell and Gutteridge, 1984). On the other hand, iron deficiency can also lead to bacterial death (Teng et al., 2018). Due to the special physical and chemical properties of iron, it is usually oxidized in an insoluble form *in vivo*, or it binds to heme, ferritin, hemoglobin, or transferrin in cells; thus, bacteria are not often exposed to iron (Teng et al., 2017). Bacteria must source sufficient iron from the host and maintain a dynamic balance for their growth. Iron plays a key role in the growth of *H. parasuis*, and low availability in a host is a primary pressure for invasion of pathogenic bacteria and is considered a signal that leads to significant changes in cell processes (Deslandes et al., 2007; He et al., 2018). In the present study, 100 μM BIP was added to fresh T/V/S medium to construct an iron-restricted environment to analyze the capability of HPS5 and Δcrp to utilize iron. The results demonstrate that the growth rates of HPS5 and Δcrp decreased when BIP was added. However, the *crp* mutant strain was more sensitive than the wild type, and the growth rates of the two strains were restored when FeSO_4_ was added (Figure 7A). We hypothesize that the *crp* gene promotes the uptake of iron from the host by regulating the expression of genes involved in iron uptake. Therefore, we measured the expression of tbpA, tbpB, and cirA, each related to iron uptake (Álvarez-Estrada et al., 2018), by real-time PCR. The results indicated that the expression of tbpA, tbpB, and cirA in the *crp* mutant were significantly reduced compared with the wild type (Figure 7B). The same result was found in *V. vulnificus*, that the growth rate declined in the iron-restricted medium by downregulating the iron uptake genes after the *crp* gene was deleted (Choi et al., 2006).

In summary, we constructed a *crp* deletion mutant from HPS5 and conducted a preliminary analysis of the effects of the *crp* gene on growth characteristics and stress resistance, including growth rate, capability to undergo autoagglutination, biofilm formation, stress resistance, serum bactericidal resistance, and iron utilization. Compared with Δcrp, we found that the *crp* gene clearly boosted growth, increased the tolerance to osmotic stress, and increased the biofilm formation capability of HPS5. Conversely, the *crp* gene caused HPS5 to become more sensitive to killing by serum and to heat shock. The *crp* gene did not affect autoagglutination or tolerance to oxidative stress in HPS5. Further studies are required to study whether the *crp* gene is related to the virulence of *H. parasuis*. The present study provides an insight into the role of the *crp* gene in the pathogenesis of *H. parasuis* infection.

## Data Availability Statement

The datasets generated for this study are available on request to the corresponding author.

## Author Contributions

CJ and QH conceptualized the study, wrote, reviewed, and edited the manuscript. CJ and YC contributed to data curation. QH was responsible for funding acquisition, project administration, and supervision. CJ, HC, and BZ worked on the investigation. CJ, HC, JL, LZ, and QH worked on the methodology. CJ, ZL, WZ, and CL were responsible for the visualization. CJ wrote the original draft.

### Conflict of Interest

The authors declare that the research was conducted in the absence of any commercial or financial relationships that could be construed as a potential conflict of interest.
