# Supplementary material for: Effect of cAMP Receptor Protein Gene on Growth Characteristics and Stress Resistance of Haemophilus parasuis Serovar 5
Source: Front Cell Infect Microbiol. 2020 Feb 25;10:19. doi: 10.3389/fcimb.2020.00019 (PMC7052058; doi:10.3389/fcimb.2020.00019)
Supplement: Supplementary file 1 [file Data_Sheet_1.docx]

***Supplementary Material***

**Table 1 The primers were used for polarity effect in this study**

| **Primers** | **Sequences** | **Function** |
| --- | --- | --- |
| **2040-F** | ATGTACCCACTTATCAAAAA | To amplify the HAPS_2040 gene (1008 bp) |
| **2040-R** | CTAAATAGAAATTTTCCTAACC |  |
| **2042-F** | ATGCACTCCTCTATTCCTTTAT | To amplify the HAPS_2042 gene (192 bp) |
| **2042-R** | TCATTTCATATTCCCAACTAAAGC |  |
| **2044-F** | ATGATTATCCCTTGGCAAG | To amplify the HAPS_2044 gene (225 bp) |
| **2044-R** | TTACAAATTCCCTAAGAAAG |  |
| **2045-F** | ATGACAGAACCTGTTATC | To amplify the HAPS_2045 gene (606 bp) |
| **2045-R** | CTAGTAGAACAATGGCTTAATC |  |

**A**


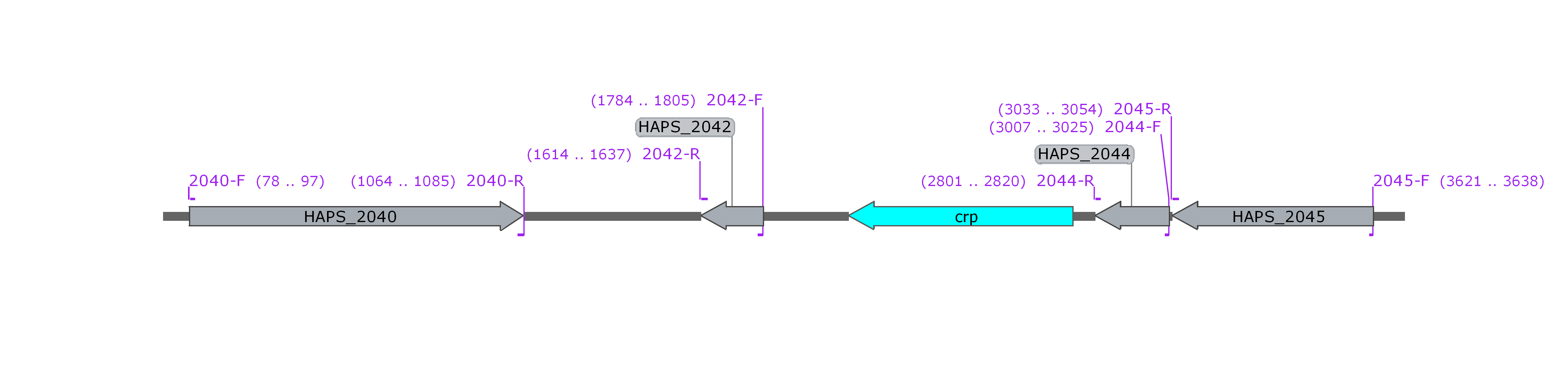


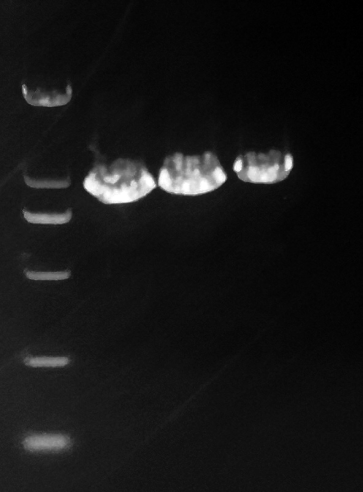


**2000bp**

**1000bp**

**750bp**

**500bp**

**250bp**

**100bp**

**M 1 2 3 N**

**HAPS_2040**


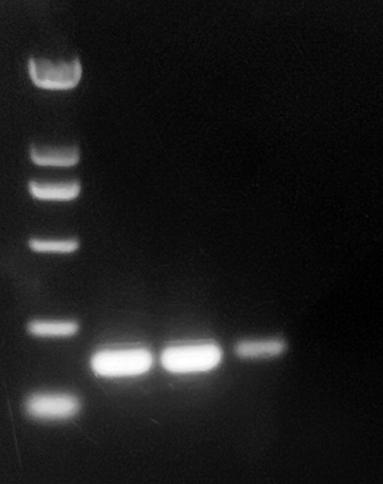


**2000bp**

**1000bp**

**750bp**

**500bp**

**250bp**

**100bp**

**M 1 2 3 N**

**HAPS_2042**


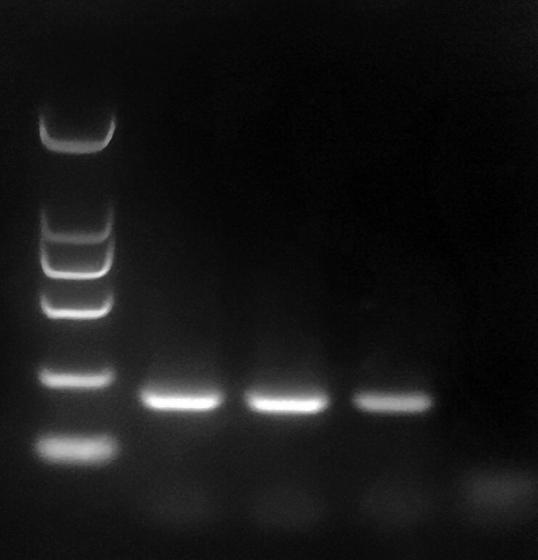


**2000bp**

**1000bp**

**750bp**

**500bp**

**250bp**

**100bp**

**M 1 2 3 N**

**HAPS_2044**

**M 1 2 3 N**


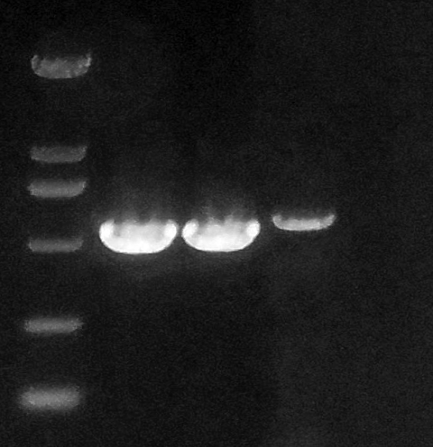


**2000bp**

**1000bp**

**750bp**

**500bp**

**250bp**

**100bp**

**HAPS_2045**

**B**

**Figure 1.** Verification the expression of the neighbor genes of the crp gene. (**A**) Distribution diagram of upstream and downstream of *crp* gene. (**B**) RT-PCR identification of the HAPS_2040, HAPS_2042, HAPS_2044 and HAPS_2045 from cDNAs of wild type and *crp* mutant strain. M: DL 2000 Mark, 1 and 2 represented cDNA genomes of the wild type and *crp* mutant, 3 represented genomes DNA (gDNA) of the wild type which was the positive control, N represented negative control.
